# Clinical features of children hospitalized with influenza A and B infections during the 2012–2013 influenza season in Italy

**DOI:** 10.1186/s12879-015-1333-x

**Published:** 2016-01-08

**Authors:** Livia Mancinelli, Manuela Onori, Carlo Concato, Roberto Sorge, Stefano Chiavelli, Luana Coltella, Umberto Raucci, Antonio Reale, Donato Menichella, Cristina Russo

**Affiliations:** 1 Virology Unit, Bambino Gesù Children’s Hospital, IRCCS, Rome, Italy; 2 Laboratory of Biometry, University of Tor Vergata, Rome, Italy; 3 Pediatric Emergency Department, Bambino Gesù Children’s Hospital, IRCCS, Rome, Italy; 4 Medical Direction, Bambino Gesù Children’s Hospital, IRCCS, Rome, Italy

**Keywords:** Influenza viruses, Viral subtypes, Children, Clinical features, Hospitalization

## Abstract

**Background:**

Influenza is a major public health issue worldwide. It is characterized by episodes of infection that involve hundreds of millions of people each year. Since that in the seasons 2010–2011 and 2011–2012 the circulation of FLUB was decreasing we evaluated the clinical presentation, demographic characteristics, admitting department, and length of stay in children who contracted influenza admitted to Bambino Gesù Children’s Hospital, during the 2012–2013 influenza season, with the aim to establish if the recover of FLUB was associated to a clinical worsening, in comparison with those due to FLUA.

**Methods:**

A total of 133 respiratory specimens, collected from patients with symptoms of respiratory tract infections, positive for the Influenza A and B viruses (FLUA and B) were subtyped. Comparisons between the FLUA and FLUB groups were performed with the one-way ANOVA for continuous parametric variables, the Mann-Whitney test for non-parametric variables, or the Chi-Square test or Fisher’s exact test (if cells <5) for categorical variables.

**Results:**

87.09 % of the FLUA isolates were the H1N1 subtype and 12.90 % were H3N2. Among the FLUB isolates, 91.54 % were the B/Yamagata/16/88 lineage and 8.45 % were the B/Victoria/02/87 lineage. The largest number of FLUA/H1N1 cases was observed in children less than 1 years old, while the B/Yamagata/16/88 lineage was most prevalent in children 3–6 years old. Fever was a common symptom for both FLUA and B affected patients. However, respiratory symptoms were more prevalent in patients affected by FLUA. The median length of stay in the hospital was 5 days for FLUA and 3 days for FLUB.

**Conclusions:**

The clinical features correlated to different Influenza viruses, and relevant subtypes, were evaluated concluding that the increasing of FLUB in the season 2012–2013 was without any dramatic change in clinical manifestation. Our findings suggest, finally, that a stronger commitment to managing patients affected by FLUA is required, as the disease is more severe than FLUB.

## Background

Influenza is one of the most common respiratory infectious diseases and is responsible for between 3 and 5 million cases of severe influenza illness each year [1]. The influenza viruses are members of the *Orthomyxoviridae* family and contain a single-stranded RNA genome that is distributed in eight separate segments. There are three types of Influenza viruses A, B and C [2] that have different structural arrangements of internal nucleoprotein and matrix protein antigens. The segmented genome may be responsible for the development of pandemic strains, whereas mutations (aminoacid substitutions) may be responsible for the development of influenza epidemics [3].

Influenza typically results in mild-to-moderate illness in healthy individuals. However, disease severity tends to increase in children, the elderly, and individuals with chronic medical conditions (pulmonary, cardiovascular, liver, renal, and neurological diseases or immunosuppression).

Children are more susceptible to infection [4] with annual incidence rates up to 30 % [5]. In particular, children under 5 years of age are more susceptible to contracting influenza since they are an immunologically naïve population [6]. Moreover, they can also be considered the primary transmitters of influenza in the community [7, 8] and shed virus at higher viral titers and for a longer period than adults [9]. Community based surveillance programs have found that the H3N2 subtype is detected more frequently in adults, while H1N1 and Influenza B viruses are detected more often in children [10].

After the beginning of the 2009 H1N1 pandemic, the Center for Disease Control (CDC) seasonal surveillance reports documented an increased prevalence of Influenza A virus (FLUA) compared to Influenza B virus (FLUB) during the 2010–2011 and 2011–2012 influenza seasons. In the 2012–2013 season FLUA and FLUB were detected in similar proportions. In Europe, the FLUA epidemic was primarily caused by the H1N1 subtype, which outnumbered the H3N2 subtype by in ratio 2:1. The FLUB epidemic was driven by the B/Yamagata lineage, which was more prevalent than the B/Victoria lineage by a ratio of 5:1 [11]. Although a large amount of data related to influenza surveillance activity is available [12–14], there are few studies that compare differences in the clinical presentations of FLUA and B subtypes isolated in children from Italy.

Since that in the seasons 2010–2011 and 2011–2012 the circulation of FLUB was decreasing, the objective of this study was to characterize the clinical features, demographic characteristics, admitting department, and length of stay in the hospital for children, affected by FLUA and FLUB, admitted to Bambino Gesù Children’s Hospital and Research Institute during the 2012–2013 FLU season, with the aim to establish if the remerging of FLUB was associated to a clinical worsening, in comparison with those due to FLUA.

## Methods

### Design of the study

A retrospective study was conducted analyzing data collected from patients resulted positive for FLU admitted to Bambino Gesù Children's Hospital and Research Institute from November 2012 to May 2013. Research ethics approval was not necessary as retrospective study in our Institution, and informed consent was not required as the data were analyzed anonymously. Patients received care in several hospital departments, but the major departments that provided care were Emergency, Pediatrics, Surgery, the Intensive Care Unit (ICU), and the Impaired Immune Function Unit (IIFU). Samples of throat and nasal swabs (44), nasopharyngeal aspirate (85), bronchoalveolar lavage (2) and sputum (2) were processed immediately or stored at −80 °C for up to two days before testing. The results of testing for the infectious agent and clinical symptoms, underlying disease, C-reactive protein (CRP), and the patient outcomes were recorded.

### Nucleic acid extraction

Viral DNA and RNA was extracted from 400 μL of specimen using the EZ1 Virus Mini Kit v. 2.0 on the EZ1 Advanced XL platform (Qiagen, GmbH, Hilden, Germany) and eluted into 60 μL of elution buffer.

### Reverse transcription

Reverse transcription was performed using Applied Biosystems (Van Allen Way, Carlsbad, California) reagents from 10 μL of extracted nucleic acids. Each reaction contained 5 μL of 10X RT buffer, 11 μL of MgCl2 (25 mM), 2.5 μL of dNTP mix (10 mM), 2.5 μL of random hexamer (50 mM), 1 μL of RNase inhibitor (20 U/mL), 1.25 μL of Reverse Transcriptase (50 U/mL), and 16.75 μL of DEPC water. The thermal cycling parameters were: 25 °C for 10 min, 42 °C for 60 min, and 95 °C for 5 min.

### Routine respiratory virus detection

A multiplex PCR panel able to identify FLU A and B, Human Metapneumovirus, Adenovirus, Coronavirus 229E/NL63, Parainfluenza viruses 1, 2, and 3, Coronavirus OC43, Rhinovirus A/B, and Respiratory syncytial A and B (RV12 ACE Detection 23 Seegene, Seoul, Korea), using the dual priming oligonucleotide (DPO) system [15], was used according to the manufacturer’s instructions. An internal control included in the primer mixtures was used to assess any potential PCR inhibitory effects. All of the samples that tested positive for FLUA or FLUB were further characterized by genotyping analysis to identify the subtype. For FLUA subtyping was performed using a procedure used in our laboratory, instead for FLUB a homebrew procedure was set-up according to WHO recommendations [16].

### Subtyping influenza A

Each FLUA positive sample was typed using Sanger sequencing analysis. The amplified products were purified using a Purelink™ Quick PCR Purification Kit (Invitrogen by Life technologies, Löhne, Germany), according to the manufacturer’s instructions. Sequencing reactions were performed using the modified standard protocol for the Big Dye Terminator Cycle Sequencing Kit 3.1 (Applied Biosystems). Briefly, each reaction contained 2 μL of big dye terminator, 2 μL of 5X big dye eluent, 1.6 μL of 1 μM FluA primers (Seegene, Seoul, Korea), 1–3.5 μL of purified amplified DNA, and water in a final volume of 10 μL. After removing the dye terminator using DyeEx 2.0 (Qiagen), sequencing analysis was performed using an ABI PRISM 3130xl Genetic Analyzer (Applied Biosystems). All of the original electropherograms were analyzed using GenBank BLAST software (http://blast.ncbi.nlm.nih.gov/Blast.cgi) to identify the FLUA subtype. Identity scores ≥99 % were considered sufficient for correctly identifying the subtype.

### Subtyping influenza B

Each FLUB positive sample was genetically characterized to discriminate between the B/Yamagata/16/88 and B/Victoria/02/87 lineages. Each PCR reaction was performed in a final volume of 50 μL and contained 10X PCR buffer (Applied Biosystems), 25 mM MgCl2, 10 mM deoxynucleoside triphosfate (dNTP), 10 μM forward primer, 10 μM reverse primer, 5 μL of template cDNA, and 250 units Amplitaq Gold polymerase. The primers used to identify the Victoria lineage were: the Bvf224 forward primer (ACATACCCTCGGCAAGAGTTTC) and the Bvr507 (TGCTGTTTTGTTGTTGTCGTTTT1) reverse primer. The primers for the Yamagata reaction were: the Byf226 forward primer (ACACCTTCTGCGAAAGCTTCA) and the BYr613 reverse primer (CATAGAGGTTCTTCATTTGGGTTT). These primers were chosen based on the recommendations of the WHO [16]. The amplified products were detected by gel electrophoresis using the Flashgel System, and 2.2 % agarose gels (Lonza, Basel, Switzerland). The expected product for the Yamagata lineage was 388 bp and 284 bp for the Victoria lineage.

### Statistical analysis

Data analysis was conducted using the Statistical Package for the Social Sciences Windows, version 15.0 software (SPSS, Chicago, Illinois, USA). The descriptive statistics consisted of the mean ± standard deviation for parameters with a Gaussian distribution, which was confirmed using histograms and the Kolgomorov-Smirnov test. The median and range (min to max) are presented for frequencies and categorical variables with non-Gaussian distributions. Comparisons between the FLUA and FLUB groups were performed with the one-way ANOVA for continuous parametric variables, the Mann-Whitney test for non-parametric variables, or the Chi-Square test or Fisher’s exact test (if cells <5) for categorical variables. A *p* value of <0.05 was considered statistically significant for all tests.

## Results

**Fig. 1:**
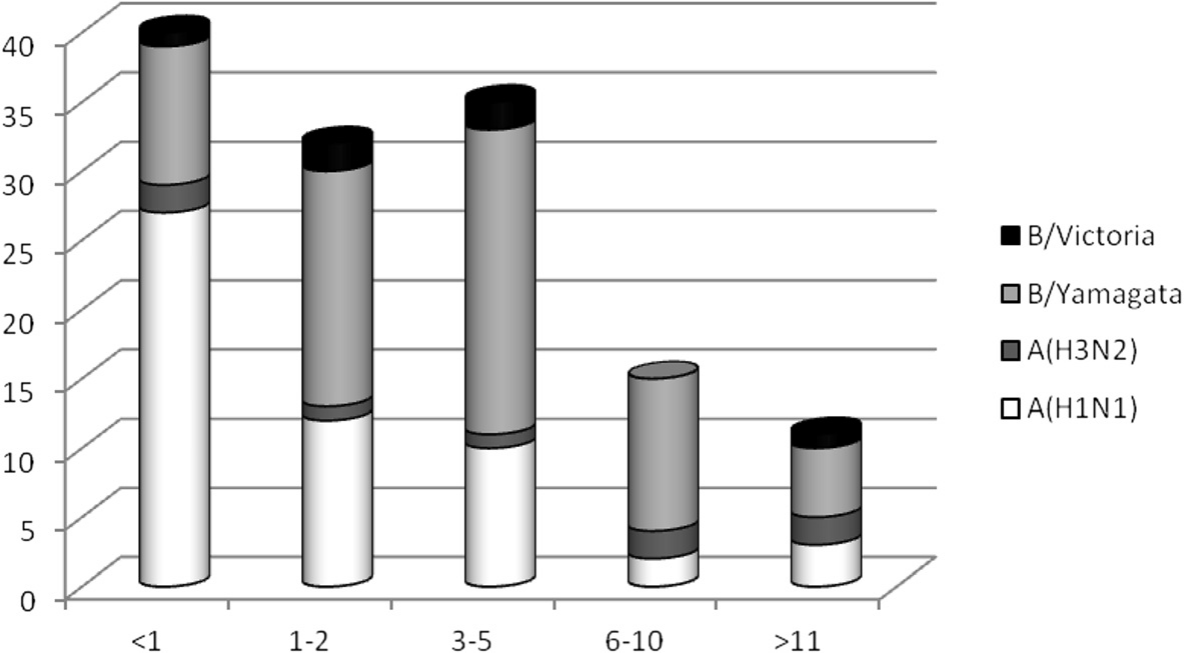
Distribution of FLUA and B in children of different ages

A total of 133 respiratory samples tested positive for FLUA (*n* = 62; 46.61 %) and FLUB (*n* = 71; 53.38 %). The most common FLUA subtype was H1N1 detected in 54/62 (87.1 %) samples while only 8/62 (12.90 %) were the H3N2 subtype. As expected, the most common FLUB subtype was the B/Yamagata/16/88 lineage detected in 65/71 (91.54 %) samples. The remaining 6/71 (8.45 %) FLUB samples were from the B/Victoria/02/87 lineage. The distribution of FLUA and FLUB patients affected by each subtype is summarized in Table 1 according to their demographic characteristics (gender, nationality and age), clinical symptoms, outcome, underlying conditions, unit of admission/hospitalization, and length of stay. Among the patients who tested positive for FLUA, 33/62 were female and 29/62 male, while for the FLUB patients 25/71 were female and 46/71 male (*p* = 0.320). All patients involved were between 0 and 16 years old. The highest number of cases of FLUA/H1N1 was observed in children less than 1 year of age. The FLUB B/Yamagata/16/88 lineage was most prevalent in children ranging from 3 to 6 years old. Infections with the less prevalent FLUA H3N2 and FLUB B/Victoria/02/87 strains were homogeneously distributed across the different age groups (Fig. 1). There were no significant differences by one-way ANOVA analysis between the FLUA and FLUB patients in terms of age (*p* = 0.370), weight (*p* = 0.203), height (*p* = 0.203), and body mass index (*p* = 0.660).

**Table 1:** Demographic and clinical characteristics of 133 patients, admitted to Bambino Gesù Children’s Hospital, Rome, Italy, during the influenza season 2012–2013, positive for FLUA and B by viral type and subtype

	FLU A		FLU B	
	H1N1 (*N* = 54)	H3N2 (*N* = 8)	Yamagata lineage (*N* = 65)	Victoria lineage (*N* = 6)
Sex	*N* (%)	*N* (%)	*N* (%)	*N* (%)
Males	26 (19.55)	3 (2.26)	42 (31.58)	4 (3.01)
Females	28 (21.05)	5 (3.76)	23 (17.29)	2 (1.50)
Nationality				
Italian	49 (36.84)	8 (6.02)	52 (39.1)	5 (3.76)
European	2 (1.50)	0	5 (3.76)	0
Other	3 (2.26)	0	8 (6.01)	1 (0.75)
Age (years)				
< 1	27 (20.3)	2 (1.50)	10 (7.52)	1 (0.75)
1–2	12 (9.02)	1 (0.75)	17 (12.79)	2 (1.50)
3–5	10 (7.52)	1 (0.75)	22 (16.55)	2 (1.50)
6–10	2 (1.50)	2 (1.50)	11 (8.28)	0
> 11	3 (2.26)	2 (1.50)	5 (3.76)	1 (0.75)
Clinical symptoms				
Fever >38 °C	37 (27.82)	4 (3.01)	27 (20.3)	4 (3.01)
Fever <38 °C	17 (12.79)	4 (3.01)	38 (28.57)	2 (1.50)
Apnea	7 (5.26)	0	2 (1.50)	0
Respiratory simtomps	29 (21.80)	3 (2.26)	21 (15.79)	3 (2.26)
Outcome				
Fever	31 (23.31)	7 (5.26)	39 (29.32)	4 (3.01)
Otitis	2 (1.50)	0	3 (2.26)	0
Laryngitis	1 (0.75)	0	1 (0.75)	0
Pharingitis	1 (0.75)	0	2 (1.50)	0
Bronchitis	4 (3.01)	0	5 (3.76)	1 (0.75)
Bronchiolitis	7 (5.26)	1 (0.75)	1 (0.75)	1 (0.75)
Bronchopneumonia	1 (0.75)	0	0	0
Pneumonia	7 (5.26)	0	11 (8.28)	0
Myositis	0	0	2 (1.50)	0
Pertussis	0	0	1 (0.75)	0
Underlying disease				
None	44 (33.08)	3 (2.26)	57 (42.86)	4 (3.01)
Acquired	2 (1.50)	2 (1.50)	7 (5.26)	0
Congenital	5 (3.76)	1 (0.75)	0 (0.00)	2 (1.50)
Genetic	3 (2.26)	2 (1.50)	1 (0.75)	0
Units				
Emergency	10 (7.52)	0	27 (20.30)	3 (2.26)
Pediatrics	30 (22.56)	4 (3.01)	24 (18.05)	1 (0.75)
Surgery	2 (1.50)	0	0	0
ICU^a^	3 (2.26)	1 (0.75)	1 (0.75)	1 (0.75)
IIFU^b^	10 (7.52)	2 (1.5)	13 (9.77)	1 (0.75)
Length of stay (days)				
Median	5	5	3	3

^a^Intensive care units

^b^Impaired immune function units

Fever was the most common symptom in FLU positive patients. In patients with FLUB a fever <38 °C (range: 37–37.9 °C) was significantly more frequent than FLUA (*p* = 0.019) and patients affected by FLUA had a significantly higher frequency of respiratory simptoms (*p* = 0.034) than patients with FLUB. Apnea was equally distributed in FLUA and FLUB patients. No significant differences were observed between the two groups in terms of laryngitis, pharyngitis, bronchitis, bronchiolitis, bronchopneumonia, pneumonia, or pertussis. Twenty five patients presented with neoplasia/hematological malignancies or a severe underlying disease, such as chronic disorders of the pulmonary or cardiovascular systems, metabolic diseases, and neurological disorders, that were clustered in congenital and genetic disorders (Table 1). In patients suffering from underlying diseases, FLUA infection was more prevalent than FLUB (*p* = 0.044). The normal concentration of CRP in healthy human serum ranges from 0 to 0.50 mg/dL. In the FLU patients, the mean CRP value was 1.71 ± 2.54 for FLUA patients and 1.14 ± 1.72 for FLUB patients. A value ≥ 0.50 was obtained in 45 % of FLUA cases and in 46 % of FLUB.

Information related to the department where patients were admitted is shown in Table 1. Patients accessed the hospital through the emergency unit in 33.7 % of FLUA and 66.3 % of FLUB cases (*p* = 0.017). The median of length of stay was 5 days (range: 0–59) for FLUA and 3 days (range: 0–116) for FLUB. Community acquired influenza infections were equally distributed between the FLUA and FLUB patients, while among nosocomial infections FLUB was responsible for 90.9 % versus 9.1 % of FLUA (*p* = 0.039).

There were no significant differences observed in the frequency of co-infections between the different types of FLU. Among the 62 patients infected with FLUA, 18 (29.03 %) co-infections with other respiratory pathogens were detected, 13 of which were co-infections with respiratory viruses and 5 were with bacteria. In 71 patients infected with Influenza B, 11 (15.5 %) co-infections were detected, 7 of which were co-infections with viruses and 4 were with bacteria. There were no co-infections detected in patients with the B/Victoria/02/87 lineage. The respiratory pathogens responsible for the co-infections are listed in Table 2.

**Table 2:** Summary of the co-infecting respiratory pathogens (viruses and bacteria) detected in FLUA and B cases

Influenza virus	Respiratory pathogens	Number (%)
FLUA (H1N1)		N/14
	Adenovirus	3 (21.43)
	Coronavirus 229E	2 (14.29)
	Parainfuenza virus 3	1 (7.14)
	Rhinovirus	1 (7.14)
	Respiratory Syncytial Virus B	2 (14.29)
	Bocavirus	1 (7.14)
	Respiratory Syncytial Virus A- Coronavirus OC43	1 (7.14)
	*Haemophylus influenzae*	2 (14.29)
	*Pseudomonas aeruginosa*	1 (7.14)
FLUA (H3N2)		N/4
	Coronavirus 229E	1 (25)
	Coronavirus 229E- Rhinovirus	1 (25)
	*Pseudomonas aeruginosa*	1 (25)
	*Branhamella catarrhalis*- *Haemophylus influenzae*- *Staphylococcus aureus*	1 (25)
FLUB Yamagata/16/88 lineage		N/11
	Coronavirus OC43	1 (9.09)
	Coronavirus 229E	1 (9.09)
	Metapneumovirus	1 (9.09)
	Rhinovirus	1 (9.09)
	Respiratory Syncytial Virus A	2 (18.19)
	Respiratory Syncytial Virus A- Adenovirus	1 (9.09)
	*Bordetella pertussis*	1 (9.09)
	*Branhamella catarrhalis*	1 (9.09)
	*Haemophylus influenzae*- *Staphylococcus aureus*	1 (9.09)
	*Streptococcus pyogenes*	1 (9.09)

**Fig. 2:**
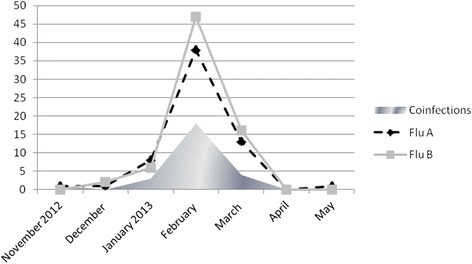
Distribution of influenza cases observed from November 2012 to May 2013

**Fig. 3:**
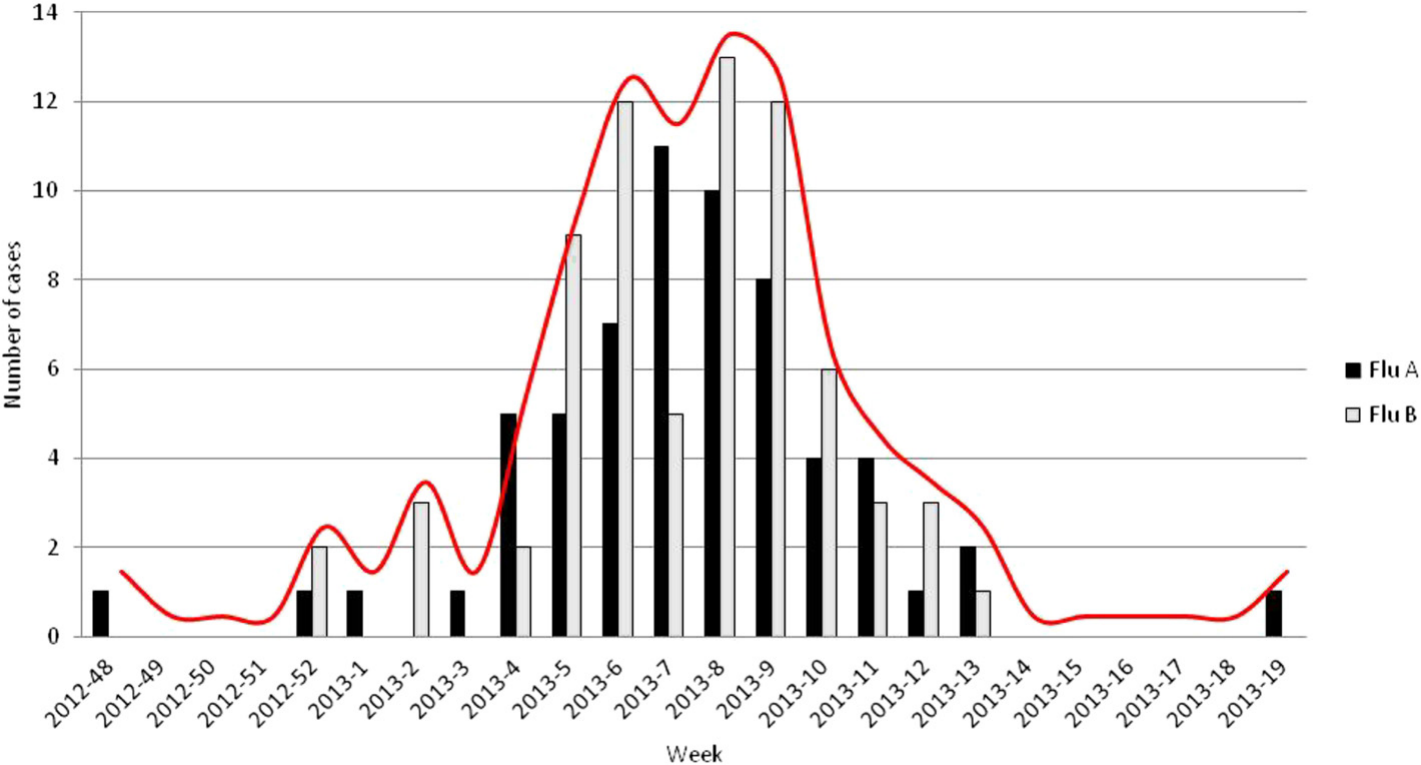
Weekly distribution of FLUA and B isolates during the 2012–2013 FLU season

The distribution of influenza cases during the 2012–2013 season is shown in Fig. 2. The peak incidence of positive cases was between the 5th and 11th week of 2013. The highest incidence occurred for FLUA and B in the 7th and 8th week, respectively, corresponding to the middle of February (Fig. 3). The first FLUA case was detected at the end of November 2012 and started circulating earlier than FLUB, which was first detected at the end of December 2012.

## Discussion

In this study, a comparison of the clinical features presented in children admitted to our hospital resulted positive for FLUA and B infections was analyzed. Bambino Gesù Children’s Hospital is a Reference Pediatric Centre for the care and treatment of children coming from central and southern Italy.

FLUB was slightly more prevalent (53.38 %) than FLUA (46.62 %) in the study population. This is consistent with the 2012–2013 ECDC Surveillance Report that reported a similar proportion of seasonal FLUA and B in Europe. However, FLUA peaked and declined slightly before FLUB and the highest infection frequency was evident during the “winter season”. Our data show a similar seasonal trend for FLUA and B; however, in contrast to the data reported by the ECDC, the circulation of FLUA ended later than FLUB.

Our results were consistent too with the prevalence of FLUA/H1N1 (34 %), FLUA/H3N2 (5 %), and FLUB (58 %) in Italy during the 2012–2013 flu season reported to Influnet, a sentinel surveillance system for influenza [17]. Influnet also reported 3 % of FLUA cases that were not subtyped. However, these data reflect the whole population and our data are concerned with only pediatric patients.

The results of laboratory testing and clinical findings were compared for FLUA and FLUB patients to investigate clinical differences between the groups. No consistent differences were observed in the clinical presentation of patients by subtype viral, according to results of studies about the clinical characteristics of patients positive for Influenza A and B [18]. Males seemed to be more susceptible to contracting FLU compared to females and, specifically, FLUB. This is concordant with previous studies in which the greater humoral and cell-mediated immune responses of females to viral antigens was demonstrated to play an important role in determining the gender variability in viral infections and, in females, being beneficial against infectious diseases [19]. With regard to the age distribution, FLUA was more common in children less than 1 year old, where they can cause more severe infections, confirming previous studies [20, 21], and FLUB was more common in school age children.

Moreover, length of stay in children with FLUA was significantly higher than those infected with FLUB, being the median 5 days (range: 0–59) for FLUA and 3 days (range: 0–116) for FLUB. Specifically, this long hospital stay (59 and 116 days) was, for FLUA, in a patient with complications due to tracheostomy procedure, while for FLUB a nosocomial infection occurred in a oncoematological patient. This finding is unsual since that in a previuos study children with influenza B in comparison with those infected by A/H1N1 influenza virus had significantly higher hospitalization rates (*p* < 0.05) [20]. Is possible to speculate that the longer hospitalization for FLUA patients be correlated to the major cases with fever >38 °C and respiratory simptoms. No significant differences were observed in CRP levels between FLUA and B patients and a similar frequency of patients with elevated CRP was detected in both FLUA and B. Fever was confirmed as a major influenza symptom [21, 22]. A similar number of FLUA and FLUB patients presented with a temperature ≥38°. However, FLUB patients were significantly more likely to have a fever <38 °C than FLUA patients. In contrast, respiratory symptoms were mainly detected in FLUA patients (Table 1). Pre-existing diseases condition the clinical expression of Influenza resulting in a greater number of these children developing lower respiratory tract infection [23]. We found that FLU patients with underlying malignancies such as lymphoma, leukemia, solid tumor, or tubulopathy, were more susceptible to FLUB infection, in agreement with data previously reported [24]. In contrast, patients with congenital diseases (hypoplastic left heart syndrome, neuromotor disorder, Swyer James syndrome, epilepsy, cerebral palsy, laryngotracheal cleft, or immune deficiency) and genetic preexisting conditions (cystic fibrosis, sickle-cell anemia, trisomy of chromosome 10, or Niemann Pick disease) were more susceptible to FLUA infection. This is probably related to FLUA patients younger than those with FLUB. In our study, 12/133 (9.02 %) of FLU infections were hospital acquired with FLUB being the principal etiological agent responsible. Patients that contracted nosocomial infections were mainly immunosuppressed and admitted through the IIFU (8 cases FLUB), followed by the pediatrics (2 cases FLUB and 1 FLUA) and surgery (1 case FLUA) units. Numerous examples of nosocomial FLU outbreaks have been reported in long-term care facilities for the elderly. Experimental evidences supports the fact that humans generate infectious particles in both respiratory droplets and aerosols and that their generation is enhanced during influenza illness [25]. Moreover, children, who do not have or minimal immunity against influenza viruses, and immunocompromised individuals, who can shed virus for long periods of time at high titers, have already been pinpointed as good transmitters in comparison to healthy adults. However, it is unclear why FLUB, in comparison to FLUA, should cause a more severe infection in the group of immunosuppressed and moreover, in the population object of the study, the number of patients with malignancies is low and it is difficult to establish if the difference is really significant.

In our study there were no significant differences between co-infections with viral or bacterial pathogens in FLUA and B patients. All of the co-infections investigated were in children with a mean age less than 5 years.

One death was reported in a FLUA patient affected by quadriplegia and chronic respiratory failure. It is possible that FLUA could have been responsible for further impeding the patient’s ability to breathe.

There were some limitations in this study. Being a retrospective study, it was not possible to collect more clinical data, as well as the information about the vaccination history of patients that tested positive for FLUA and B; in addition, this was a single-center study and only one year was analysed.

Our study was primarily focused on the clinical presentation of FLUA and FLUB infections to provide additional information concerning the clinical presentation of pediatric influenza. No outcomes due to other respiratory viruses were evaluated. Further studies that describe how co infections with other viruses impact FLU infections could be useful. To our knowledge, there is limited published data regarding the clinical differences between seasonal FLUA and B in pediatrics after the 2009 pandemics. The main findings from our study confirm that, although fever is a major component of influenza A and B presentation, respiratory symptoms were more severe and the length of the hospital stay was longer for FLUA patients than FLUB patients concluding that the increasing of FLUB in the season 2012–2013 was without any dramatic change in clinical manifestation. Although some studies have reported gastrointestinal symptoms such as abdominal pain, diarrhea and vomiting to be more common with FLUB infection [26], this was not the case in this study. The different viral types and subtypes should be routinely identifed by diagnostic laboratory to best address clinicians to appropiate therapeutic measures.

## Conclusions

In summary, our results suggest that the clinical features correlated to different FLU viruses and to the relevant subtypes should be taken into consideration by health authorities to implement prevention strategies with the aim to reduce the number of sick subjects, the prevalence of hospitalization, and the circulation of FLU.

## Abbreviations

CRP: c-reactive proteine
DPO: dual priming oligonucleotide
FLUA: influenza A virus
FLUB: Influenza B virus
ICU: intensive care unit
IIFU: impaired immune function unit
